# On-admission serum uric acid predicts outcomes after acute myocardial infarction: systematic review and meta-analysis of prognostic studies

**DOI:** 10.3325/cmj.2012.53.162

**Published:** 2012-04

**Authors:** Vladimir Trkulja, Siniša Car

**Affiliations:** 1 Department of Pharmacology, University of Zagreb School of Medicine, Zagreb, Croatia; 2 Cardiology unit, Department of Internal Medicine, General Hospital Varaždin, Varaždin, Croatia

## Abstract

**Aim:**

To evaluate the prognostic value of serum uric acid (SUA) in acute myocardial infarction (AMI) patients.

**Methods:**

Systematic review and random-effects meta-analysis of prognostic studies assessing AMI outcomes (death, major adverse cardiac events, MACE) in relation to on-admission SUA.

**Results:**

Nine studies (7655 patients) were identified, 6 in the ST-segment elevation AMI patients treated with invasive revascularization and three in mixed AMI type cohorts with variable reperfusion strategies. “High” SUA (vs “low,” different cut-offs) was univariately associated with higher short-term mortality (8 studies/6805 patients; odds ratio [OR], 3.24; 95% confidence interval [CI], 2.47-4.27) and incidence of MACE (7/6467; OR, 2.46; 95% CI, 1.84-3.27, moderate heterogeneity, mild bias), and with higher medium-term mortality (5/5194; OR, 2.69; 95% CI, 2.00-3.62, moderate heterogeneity, mild bias) and MACE (4/4299; OR, 1.93; 95% CI, 1.36-2.74, high heterogeneity, mild bias). It was independently associated with a higher short-term (4/3625; OR, 2.26, 95% CI, 1.85-2.77) and medium/long-term (3/2683; hazard ratio [HR], 1.30; 95% CI 1.01-1.68, moderate heterogeneity, mild bias) occurrence of poor outcomes (death/MACE). As a continuous variable (by 50 μmol/L), higher SUA was also independently associated with poorer medium/long-term outcomes (4/3533; HR, 1.19; 95% CI, 1.03-1.37, high heterogeneity, mild bias). All individual study effects (unadjusted or adjusted) were in the same direction, but differed in size. Heterogeneity was mainly due to the included AMI type and/or definition of MACE. All bias-corrected pooled effects remained significant.

**Conclusion:**

Based on the available data, high(er) on-admission SUA independently predicts worse short-term and medium/long-term outcomes after AMI. However, the number of data are modest and additional prospective studies are warranted.

In humans, uric acid is the end product of purine metabolism due to a genetically determined lack of uricase activity (1). It is generated by oxidation of xanthine, primarily in the liver, gut, kidneys, and apparently in the heart, but xanthine oxidase (XO) is a ubiquitous enzyme (2). Serum levels of uric acid (serum uric acid, SUA) are governed by its production and elimination rates (via the kidney). Concentrations >420 μmol/L in men and >360 μmol/L in women are conventionally considered to represent hyperuricemia, values <310 μmol/L and <250 μmol/L, respectively, are considered low-normal, whereas concentrations in-between these limits are considered high-normal (3). High purine intake (eg, animal foods, herring, anchovies, alcohol, fructose, sweetbreads) and a number of morbidity (reduced renal function, conditions with a high cellular turnover), pharmacological (eg, diuretics) and genetically determined factors (eg, urate transporter or organic anion transporter mutations) may contribute to development of high SUA (4).

Uric acid has several effects of potential interest in cardiovascular diseases (CVD). It is a potent antioxidant but can also promote oxidative stress, particularly at high concentrations and/or in surroundings with a low pH and/or low levels of other antioxidants (3-5). Furthermore, in vitro*,* it has several effects on the vascular smooth muscle and mononuclear cells that are considered important in pathophysiology of CVD (5). Consequently, high(er) SUA has been extensively evaluated as a prognostic factor for different CVDs (3-5). However, increased SUA is linked to various conditions that per se are CVD risk factors (eg, hypertension, dyslipidemia, diabetes, metabolic syndrome, renal failure) and it has not been always possible to distinguish whether it is a cause or a consequence of such conditions (3-5). Next, in a failing heart or a hypoxic heart, activation of XO occurs (2,6). This inevitably results in increased SUA, but XO per se promotes oxidative stress and endothelial dysfunction (2). Inhibition of XO re-establishes endothelial function, whereas lowering of SUA by uricosuric agents does not seem to achieve this effect (2). Hence, the role of SUA in CVD has been accompanied by a controversy: should it be viewed as a “true” risk factor (ie, a “direct pathogen”) or as a mere marker of conditions that actually are the risk factors (6).

Considering coronary artery disease, a recent meta-analysis of 26 large prospective cohort studies indicated an independent association between hyperuricemia and occurrence of the disease and related mortality (7). Less is known about SUA as a potential predictor of outcomes in patients affected by the acute myocardial infarction (AMI). By 2009, two studies indicated independent association between high(er) on-admission SUA and worse outcomes (8,9). The aim of the current study was to perform a systematic review and, if feasible, meta-analysis of observational studies in order to evaluate the prognostic value of SUA in this setting.

## Materials and methods

### Literature search and study eligibility

We searched PubMed Medline, Ovid Medline, and Embase (till February 2012, no language restrictions; Web extra material 1) and bibliographies of studies selected for full-text retrieval. Considered were only prognostic studies (prospective or retrospective) that assessed major AMI outcomes (death or major adverse cardiac events, MACE), irrespectively of the post-index event observational period, AMI type (with, STEMI, or without, NSTEMI, ST-segment elevation), and treatment strategy, specifically in relation to on-admission SUA.

### Study selection and abstracting

The two authors independently reviewed the titles, abstracts, and full-text articles to decide on study inclusion and also abstracted the selected studies. Disagreements and uncertainties were resolved by a consensus.

### Outcomes

The outcomes of interest were mortality and incidence of MACE. Expectedly, definitions of MACE differed across studies, but always included all or most of the events such as re-infarction, angina, need for revascularization, cardiac arrest, death. Therefore, data for this composite outcome were used as reported. Extracted were time-to-event data, as well as “single time-point” data. Data were extracted for SUA as a continuous variable or as a categorical variable, eg, “high” vs “low” SUA irrespectively of the cut-off values used to define the categories.

### Data adjustments

Raw data on a previously described cohort (9) were available and were re-calculated to fit the format suitable for data pooling (Web extra material 2). Published data only were used for all other studies. Effect measures for SUA as a continuous variable expressed by different “steps” in SUA increase (eg, by 1, 10 or 100 μmol/L or by 1 mg/dL) were converted to SI units (μmol/L, 1 mg/dL = 59.48 μmol/L) and expressed per 50 μmol/L. For data adjustments see Web extra material 2.

### Data pooling and statistical analysis

Outcomes reported at individual time points were grouped into two time intervals after AMI. The short-term interval referred to the in-hospital period or, if data not available, to the first 30 days after AMI (in-hospital data were preferred assuming greater reliability). Medium-term interval referred to the outcomes reported at 1 year after AMI, or alternatively at 6 months to two years after AMI. Pooled estimates were generated for unadjusted and independent (from multivariate analysis) SUA effects for each outcome/time interval available from ≥3 studies. Estimation of unadjusted effects of “high” vs “low” SUA was based on reported frequencies. Since some studies were declared as retrospective and some as prospective, both odds ratios (OR) and risk ratios (RR) were calculated. Estimation of the effects of SUA as a continuous variable and estimation of all independent effects was based on ln(OR). For the time-to-event data, pooled estimates were obtained only for independent effects based on ln(hazard ratio, HR). Considering the differences in evaluated settings (STEMI/NSTEMI patients combined, only STEMI patients, different reperfusion strategies, different covariate adjustments), random-effects (DerSimonian-Laird) meta-analysis was performed. Since the number of studies was small, for assessment of bias we combined visual inspection of the funnel plots, position of the 95% confidence intervals around the intercept in Egger’s regression and Duval and Tweedie’s trim and fill method. Heterogeneity (based on I^2^ and Q test) was explored only in relation to categorical moderators. Mixed-effect approach was used to analyze the subsets and to generate the across-subset estimates. We used CMA software version 2.2 (Biostat Inc., Englewood, NJ, USA).

## Results

### Characteristics of the selected studies

**Figure 1:**
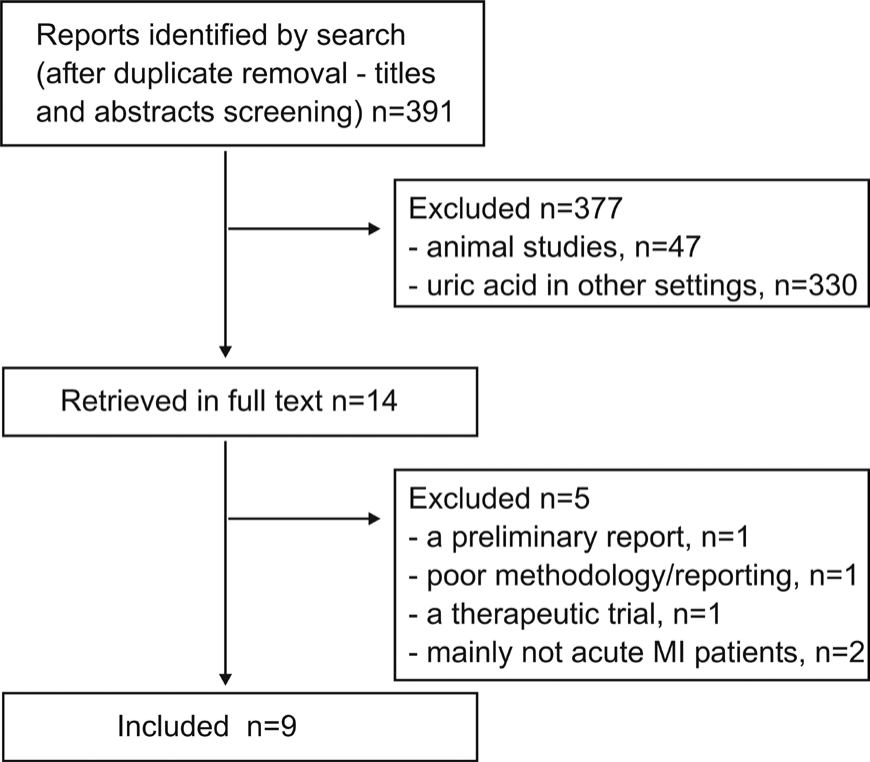
Study selection flow. MI – myocardial infarction.

Of the 391 identified individual study reports, 14 studies were retrieved in full text and 9 (7655 patients) met the inclusion criteria (Figure 1, Table 1, descriptions in the Web extra material 1). Three studies included mixed STEMI/NSTEMI cohorts with variable reperfusion strategies (8,9,14), whereas six studies included exclusively STEMI patients who underwent primary percutaneous coronary intervention (PCI) (10-13,15,16) (Table 1). The reported outcomes were short-term (in-hospital, 30-day) or medium/long-term outcomes with variably long follow-up periods (Table 1). In all studies and for all reported outcomes, high(er) on-admission SUA was univariately associated with worse results (Table 2). All studies reported also on independent associations between SUA and at least one of the assessed outcomes (Table 2) (Web extra material 1 for multivariate models). High(er) SUA was independently associated with worse outcomes in all studies (Table 2). In one study (12), it was independently associated with higher in-hospital incidence of MACE but not with in-hospital mortality (Table 2).

**Table 1:** General characteristics of studies (in chronological order) evaluating the prognostic value of on-admission serum uric acid (SUA) for outcomes of the acute myocardial infarction (AMI) included in the present analysis. For detailed descriptions, see Web extra material 1*

Study/type	Data source	Patients	Outcomes and analysis approach to medium & long-term data
Kojima et al 2005 (8) Retrospective	Japan, multicenter, admission period 2002	Consecutive AMI, type not specified; admission ≤48 h since onset; N = 1124; Reperfusion (likely STEMI) n = 943 (84%): PCI 889, thrombolysis 54; no reperfusion (some likely NSTEMI) n = 181 (16%)	Short-term: 30-d mortality and MACE (cardiac death, re-infarction, unstable angina, heart failure, stroke) Medium & long-term: mortality, maximum follow-up 699 d Time-to-event data (proportional hazard regression)
Car&Trkulja 2009 (9) Retrospective	Croatia, single center, admission period 1996-2001	Consecutive AMI; admission ≤48 h since onset; N = 621, STEMI n = 481 (77.5%), NSTEMI n = 140; Reperfusion: 10% of STEMI (thrombolysis)	Short-term: in-hospital and 30-d mortality Medium & long-term: mortality, maximum follow-up 13 y; n = 544 who survived the first 30 d Time-to-event data (proportional hazard regression)
Lazzeri et al 2010 (10) Prospective	Italy, single center, admission period likely 2004-2005^†^	Consecutive STEMI/PCI within 12 h since onset, N = 466	Short-term: in-hospital mortality
Kowalczyk et al 2010 (11) Retrospective	Poland, single center, admission period 2000-2007	Consecutive STEMI/PCI (likely within 12 h since onset) with reduced renal function – either on admission or caused by PCI, N = 1015	Mortality; MACE (death, AMI, repeated PCI, CABG, stroke) Short-term: in-hospital and 30-d Medium & long term: at 1 y and remote, maximum follow-up 93 mo Time-to-event data (proportional hazard regression)
Lazzeri et al 2011 (12) Prospective	Italy, single center, admission period 2005-2009^†^	Consecutive STEMI/PCI within 12 h since onset, N = 856	Short-term: in-hospital mortality and MACE (pulmonary edema, arrhythmia)
Basar et al 2011 (13) Prospective	Turkey, single center, admission period unspecified	Consecutive STEMI/PCI within 12 h since onset, N = 185	Short-term: in-hospital mortality and MACE (death, AMI, repeated revascularization) Medium & long-term: mortality at 1 y Single time point binary data (logistic regression)
Bae et al 2011 (14) Retrospective	South Korea, single center, admission period 2005-2008	Consecutive AMI, N = 850, STEMI n = 391 (46%), other not specified (likely NSTEMI); Reperfusion: 623 PCI (73.3%), other not specified; Onset-admission time not specified	Medium & long-term: MACE (death, non-fatal AMI or repeated PCI), follow-up 6 mo Time-to-event data (proportional hazard regression)
Akpek et al 2011 (15) Retrospective	Turkey, single center, admission period 2006-2010^‡^	Consecutive STEMI/PCI within 6 h since onset, N = 289	Short-term: in-hospital mortality and MACE (death, non-fatal AMI, stent thrombosis)
Kaya et al 2012 (16) Retrospective	Turkey, likely multicenter, admission period 2003-2009^‡^	Consecutive STEMI/PCI likely within 6 or 12 h since onset, N = 2249	Short-term: in-hospital MACE (death, re-infarction, repeated PCI, stent thrombosis) Medium & long-term: MACE (*cardiovascular death*^§^ or other events as above), maximum follow-up 55 mo Single time point binary data (logistic regression)

*Abbreviations: CABG – coronary artery by-pass grafting, MACE – major adverse cardiac events, NSTEMI – myocardial infarction without ST-segment elevation, PCI – percutaneous coronary intervention, STEMI – myocardial infarction with ST-segment elevation.

†Two reports by the same group stated to be completely separate cohorts.

‡Partial overlap of patients in the two reports is possible: the admission periods overlapped and the authors of the earlier smaller report (15) are listed among the authors of the later larger report (16).

§No description given about the methods of identification of specific cardiovascular deaths.

**Table 2:** Associations of on-admission serum uric acid (SUA) concentration with outcomes after acute myocardial infarction as reported in studies depicted in Table 1. For multivariate models in each study, see Web extra material 1*

Study	SUA (μmol/L) as	Univariate associations (95% CI)	Independent associations (95% CI)
Kojima et al 2005 (8)	Binary: “high” (4th quartile, >399, n = 276) vs “low” (1st quartile, <274, n = 274) Continuous: by 1	30-d mortality High 11% vs low 2%, *P* < 0.001 30-d MACE^†^ High 14% vs low 5%, *P* < 0.001	Mortality over 699 d High vs low: HR, 3.716 (1.417-9.741) Continuous: HR, 1.004 (1.002-1.006)
Car&Trkulja 2009 (9)	Continuous: by 10	In-hospital mortality RR, 1.038 (1.026-1.051) 30-d mortality RR, 1.035 (1.024-1.047) All-cause mortality over 13 y HR, 1.027 (1.015-1.039)	In-hospital mortality RR, 1.016 (1.001-1.031) 30-d mortality RR, 1.016 (1.003-1.029) Mortality over 13 y HR, 1.105 (1.020-1.195)
Lazzeri et al 2010 (10)	Binary: “high” (n = 100) vs “low” (n = 366), cut-off 387	In-hospital mortality High 9.0% vs low 2.5%, *P* = 0.006	In-hospital mortality OR, 2.02 (1.47-2.78)
Kowalczyk et al 2010 (11)	Binary: “high” (n = 352) vs “low” (n = 663), cut-off 420 Continuous: by 100	In-hospital mortality High 14.5% vs low 7.1%, *P* < 0.001 30-d mortality High 16.9% vs low 7.7%, *P* < 0.001 30-d MACE^†^ High 19.3% vs low 9.8%, *P* < 0.001 All-cause mortality at 1 y High 25.0% vs low 13.7%, *P* < 0.001 MACE^†^ at 1 y High 47.2% vs low 40.4%, *P* = 0.040	Mortality over 93 mo High vs low: HR, 1.17 (1.05-1.29) Continuous: HR, 1.08 (1.04-1.12)
Lazzeri et al 2011 (12)	Continuous: by 59.48 (1 mg/dL)	In-hospital mortality OR, 1.24 (1.03-1.51) In-hospital MACE OR, 1.16 (1.06-1.26)	In-hospital mortality OR, 1.02 (0.83-1.26) In-hospital MACE OR, 1.11 (1.01-1.21)
Basar et al 2011 (13)	Binary: “high” (n = 45) vs “low” (n = 140), cut-off 387 Continuous: by 59.48 (1 mg/dL)	In-hospital mortality High 6.6% vs low 2.8%, *P* < 0.01 In-hospital MACE^†^ High 11.1% vs low 5.7%, *P* < 0.01 All cause mortality at 1 y High 11.1% vs low 5.7%, *P* < 0.01 Continuous: OR, 1.21 (1.05-1.29) MACE^†^ at 1 y High 17.7% vs low 10.0%, *P* = 0.017	Mortality at 1 y High vs low: OR, 1.16 (1.10-1.41) Continuous: OR, 1.10 (1.04-1.22)
Bae et al 2011 (14)	Continuous: by 59.48 (1 mg/dL) Binary: cut-off 419, not reported for all^‡^	—	MACE^†^ over 6 mo Continuous: HR, 1.297 (1.075-1.565)
Akpek et al 2011 (15)	Binary: “high” (n = 148) vs “low” (n = 141), cut-off 321	In-hospital mortality High 13.0% vs low 2.0%, *P* < 0.001 In-hospital MACE^‡^ High 26% vs low 6.0%, *P* < 0.001	In-hospital MACE^†^ OR, 2.75 (1.93-3.94)
Kaya et al 2012 (16)	Binary: “high” (n = 606) vs “low” (n = 1643), cut off 416 for men, 356 for women	In-hospital mortality High 9.0% vs low 2.0%, *P* < 0.001 In-hospital MACE^†^ High 16.0% vs low 7.0%, *P* < 0.001 Cardiovascular mortality during follow-up High 10.0% vs low 4.0%, *P* < 0.001 MACE^†^ during follow-up High 41.0% vs low 26%, *P* < 0.001	In-hospital MACE^†^ OR, 2.03 (1.25-3.75) MACE^†^ during follow-up OR, 1.64 (1.05-2.56)

*Abbreviations: CI – confidence interval, HR – hazard ratio, OR – odds ratio, RR – relative risk (risk ratio).

†MACE (major adverse cardiac events) includes death. Definitions by study are depicted in Table 1.

‡Primary analysis was not in respect to “high” or “low” SUA. However, incidence of MACE at 30 d could be derived for 749 patients classified based on this cut-off, and incidence of MACE for “high” vs “low” at 6 mo could be derived for all 850 patients.

Of the five excluded studies (3411 patients, Web extra material 1), one (17) reported on a subset of patients from one of the included studies, another one (18) was poor by methodology/reporting (both indicated univariate association between higher on-admission SUA and worse in-hospital AMI outcomes), one was a small randomized controlled trial (19) indicating beneficial effects of allopurinol (an XO inhibitor) on short-term biochemical and electrophysiological indicators in STEMI/PCI patients, and two (20,21) were prognostic studies in patients undergoing revascularization procedures, but only a minority were AMI patients. One indicated independent association of “high” SUA and higher long-term all-cause mortality (20), and the other one reported independent association of “high” SUA and occurrence of PCI-induced renal impairment (21).

### Pooled estimates of univariate associations

**Figure 2:**
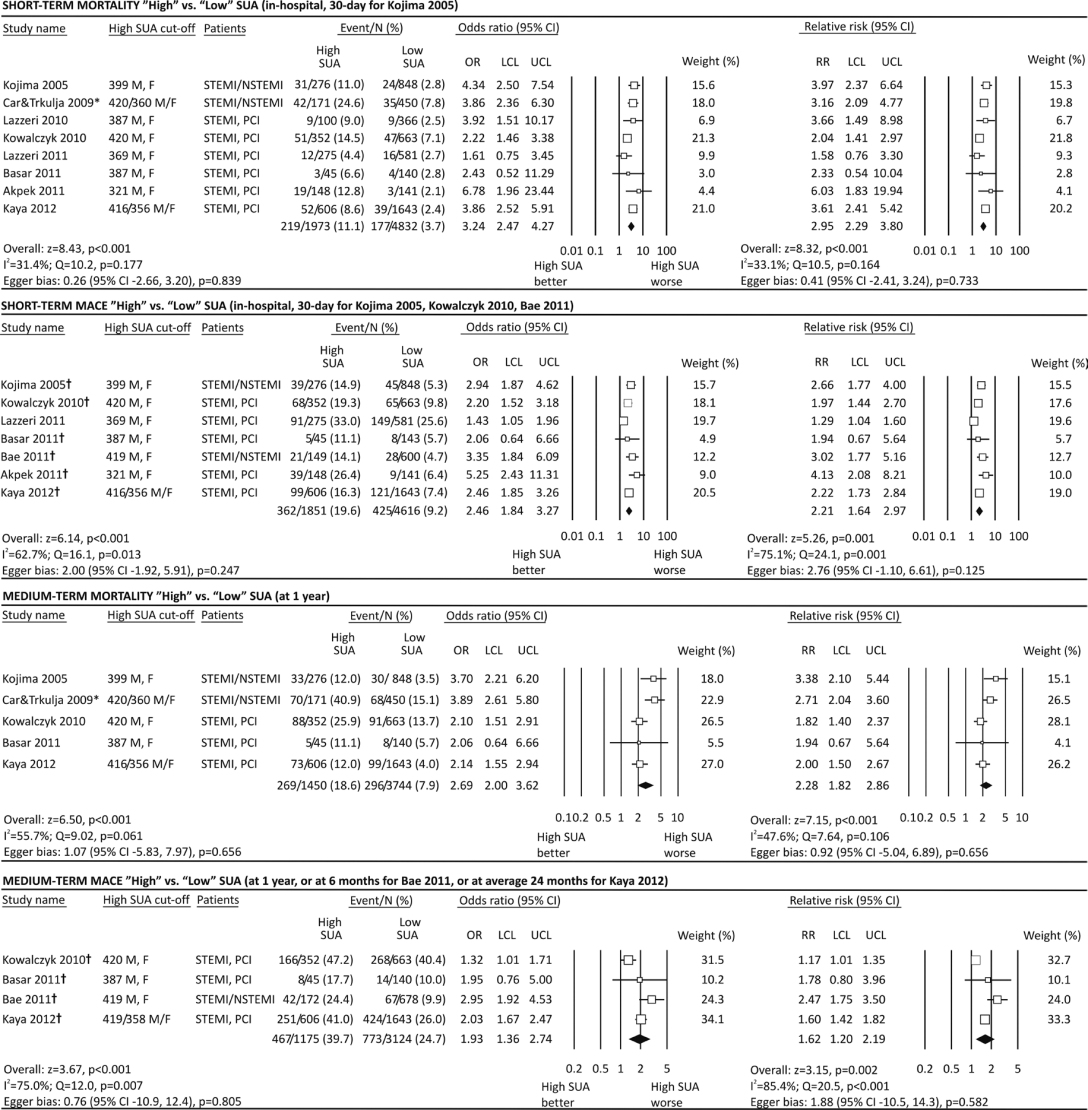
Random-effects meta-analysis of unadjusted (univariate) effects of on-admission serum uric acid (SUA) on different outcomes after acute myocardial infarction (AMI). The effects are expressed as a contrast between “high” (above a defined cut-off value, in μmol/L) and “low” (below the cut-off) SUA. Asterisk represents original cohort data recalculated (see text and Web extra material 1). Dagger indicates when death is included. M – men, F – women, STEMI – AMI with ST-segment elevation, NSTEMI – AMI without ST-segment elevation, PCI – percutaneous coronary intervention.

*Short-term mortality.* Based on eight studies (6805 patients), two in mixed STEMI/NSTEMI cohorts (8,9) and six in STEMI/PCI patients (10-13,15,16), “high” SUA was associated with higher mortality – OR, 3.24 (95% CI, 2.47-4.27), RR, 2.95 (95% CI, 2.29-3.80) – with mild heterogeneity (Figure 2) and no indication of bias. After removal of the study by Akpek 2011 (15) (low SUA cut-off, potential partial patient overlap with the later larger study by Kaya 2012 [16]), the effect in five studies in STEMI/PCI patients (OR, 2.72; 95% CI, 1.91-3.87; I^2^ = 31.9%) was somewhat lower than the effect in studies in mixed STEMI/NSTEMI cohorts (OR, 4.07; 95% CI, 2.82-5.87; I^2^ = 0.0%); between-subset Q = 2.40, df = 1, *P* = 0.121.

*Short-term incidence of MACE.* Based on seven studies (6467 patients), two in mixed STEMI/NSTEMI cohorts (8,14) and five in STEMI/PCI patients (11-13,15,16), “high” SUA was associated with higher incidence of MACE – OR, 2.46 (95% CI, 1.84-3.27), RR, 2.21 (95% CI, 1.64-2.97) – with moderate to high heterogeneity (Figure 2). The confidence intervals around the Egger’s regression intercept (Figure 2), inspection of the funnel plot, and the trim and fill method (not shown) indicated bias, ie, “a missing study” on the left hand-side of the funnel plot. The bias-adjusted effect was not much changed: OR, 2.28 (95% CI, 1.70-3.07). When the study by Akpek 2011 (15) was excluded (low SUA cut-off, potential patient overlap), the effect in four studies in STEMI/PCI patients (OR, 1.98; 95% CI, 1.48-2.67; I^2^ = 54.7%) was lower than the effect in two studies in mixed STEMI/NSTEMI cohorts (OR, 3.08; 95% CI, 2.15-4.42; I^2^ = 0.0%); between-subset Q = 3.41, df = 1, *P* = 0.065. Also, the effect in three STEMI/PCI studies in which MACE included death (OR, 2.35; 95% CI, 1.88-2.93; I^2^ = 0.0%) was higher than the effect in the single study in which death was not included in this composite outcome (Figure 2) (between-subset Q = 6.37, df = 1, *P* = 0.012). The across subset (STEMI/NSTEMI, STEMI/PCI with death included, STEMI/PCI death not included) effect was OR, 2.17 (95% CI, 1.85-2.55), Q = 10.9, df = 2, *P* = 0.004. Therefore, the among-study heterogeneity was likely attributable to the type of AMI included and (non)inclusion of death into the composite MACE outcome.

*Medium-term mortality.* Based on five studies (5194 patients), two in mixed STEMI/NSTEMI cohorts (8,9) and three in STEMI/PCI patients (11,13,16), “high” SUA was associated with higher mortality – OR, 2.69 (95% CI, 2.00-3.62); RR, 2.28 (95% CI, 1.82-2.86) – with moderate heterogeneity (Figure 2) and a slight indication of bias, ie, “a missing study” on the left hand-side of the funnel plot. The bias-adjusted effect was not much changed: OR, 2.40 (95% CI, 1.74-3.30). The effect in studies in STEMI/NSTEMI patients (OR, 3.82; 95% CI, 2.79-5.24; I^2^ = 0.0%) was significantly higher than the effect in studies in STEMI/PCI patients (OR, 2.11; 95% CI, 1.69-2.65; I^2^ = 0.0%): between-subset Q = 8.99, df = 1, *P* = 0.003; pooled estimate across the subsets: OR, 2.58 (95%, 2.15-3.09). Therefore, the among-study heterogeneity was likely attributable to the type of AMI included.

*Medium-term incidence of MACE.* Based on four studies (4299 patients), one in a mixed STEMI/NSTEMI cohort (14) and three in STEMI/PCI patients (11,13,16), “high” SUA was associated with higher incidence of MACE – OR, 1.93 (95% CI, 1.36-2.74); RR, 1.62 (95% CI, 1.20-2.19) – with high heterogeneity (Figure 2) and a slight indication of bias, ie, “a missing study” on the left hand-side of the funnel plot. The bias-adjusted effect was not much changed: OR, 1.69 (95% CI, 1.18-2.42). The effect in studies in STEMI/PCI patients was lower (OR, 1.68; 95% CI, 1.18-2.41) than the effect in the single study in STEMI/NSTEMI patients (Figure 2) (between-subset Q = 3.83, df = 1, *P* = 0.050), but among-study heterogeneity in the STEMI/PCI studies was also considerable (I^2^ = 71.0%).

### Pooled estimates of independent associations

**Figure 3:**
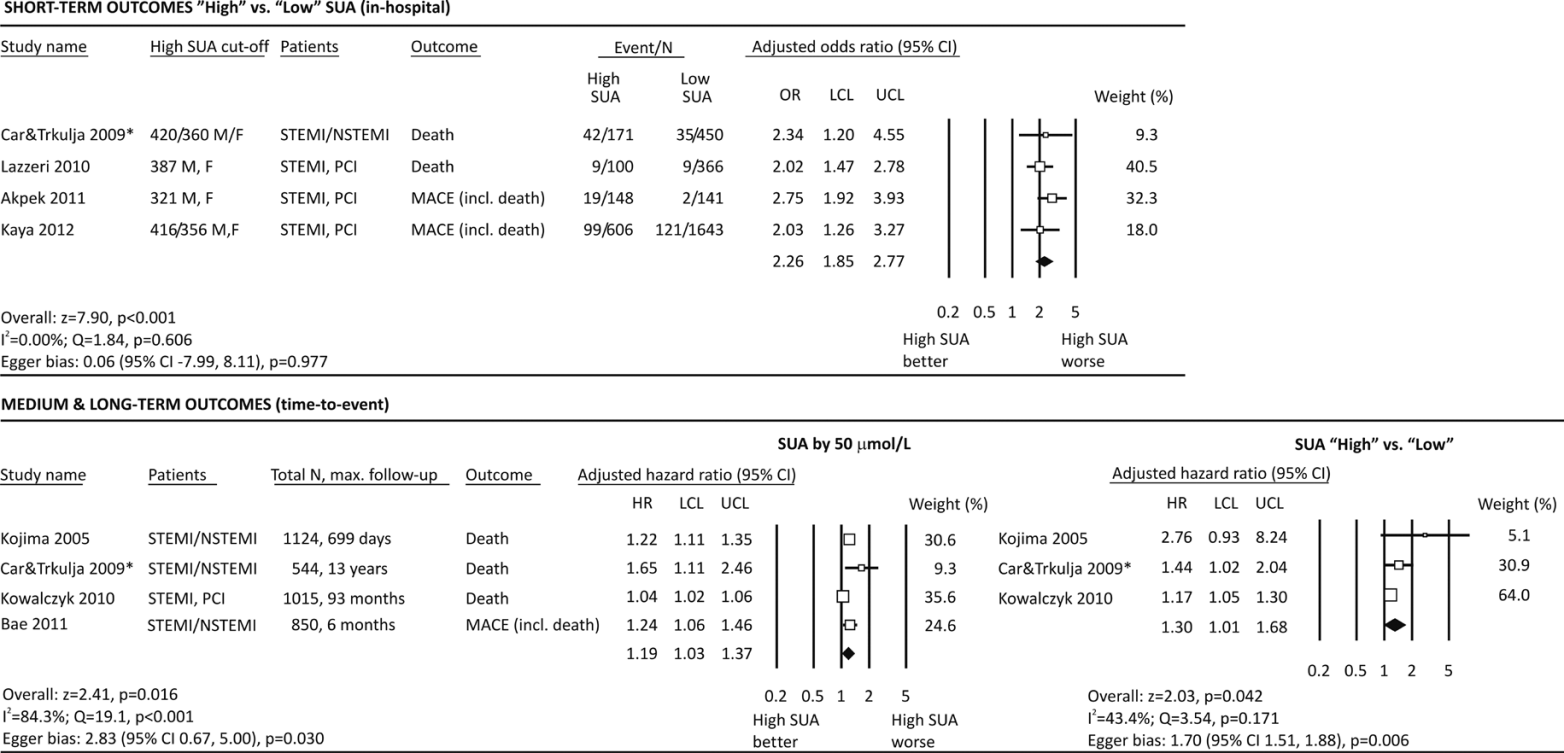
Random-effects meta-analysis of independent effects of on-admission serum uric acid (SUA) on different outcomes after acute myocardial infarction (AMI). The effects are expressed as a contrast between “high” (above a defined cut-off value, in μmol/L) and “low” (below the cut-off) SUA, or by 50 μmol/L increase in SUA concentrations. Asterisk indicates original cohort data recalculated (see text and Web extra material 1). M – men, F – women, STEMI – AMI with ST-segment elevation, NSTEMI – AMI without ST-segment elevation, PCI – percutaneous coronary intervention.

*Short-term outcomes (in-hospital).* Based on four studies (3625 patients), one in a mixed STEMI/NSTEMI cohort (9) and three in STEMI/PCI patients (10,15,16), “high” SUA was independently associated with higher incidence of adverse outcomes (death or MACE including death) – OR, 2.26 (95% CI, 1.85-2.77) – with no heterogeneity (Figure 3) and no indication of bias. The estimate was not much changed after removal of the study by Akpek 2011 (15) (low SUA cut-off, potential patient overlap): OR, 2.06 (95% CI, 1.61-2.64); I^2^ = 0.0%.

*Medium & long-term outcomes (as time-to-event).* Based on four studies (3533 patients), three in mixed STEMI/NSTEMI cohorts (8,9,14) and one in STEMI/PCI patients (11), higher SUA (by 50 μmol/L) was independently associated with higher mortality – HR, 1.19 (95% CI, 1.03-1.37) – with high heterogeneity (Figure 3) and bias, ie, two “missing studies” on the left hand-side of the funnel plot. The bias-adjusted effect was not much changed: HR, 1.15 (95% CI, 1.01-1.32). The effect in studies in STEMI/NSTEMI patients (HR, 1.24; 95% CI, 1.14-1.35; I^2^ = 3.9%) was significantly higher than the effect in the single study in STEMI/PCI patients (Figure 3): between-subset Q = 15.8, df = 1, *P* < 0.001; pooled estimate across the subsets: HR, 1.05 (95% CI, 1.03-1.07). Therefore, heterogeneity among the studies was likely attributable to the type of AMI included.

Based on three studies (2683 patients), two in mixed STEMI/NSTEMI cohorts (8,9) and one in STEMI/PCI patients (11), “high” SUA was independently associated with higher mortality – HR, 1.30 (95% CI, 1.01-1.68) – with moderate heterogeneity (Figure 3). When data for Kojima 2005 (8) were used as reported (4th vs 1st quartile patients), the pooled estimate was not much changed, but precision was reduced and heterogeneity increased (HR, 1.45; 95% CI, 0.99-2.11; I^2^ = 69.5%). The random-effect estimate adjusted for bias was reduced: HR, 1.17 (95% CI, 0.93-1.48). The overall fixed-effect estimate was HR, 1.20 (95%, 1.09-1.32) and was not much changed when adjusted for bias: HR, 1.17 (95%, 1.06-1.29). The effect in two studies in STEMI/NSTEMI patients (OR, 1.53; 95% CI, 1.10-2.13; I^2^ = 19.7%) was somewhat greater than the effect in the single study in STEMI/PCI patients (Figure 3).

## Discussion

The role of SUA as a potential risk predictor of outcomes in AMI has been more closely investigated only recently: 8/9 studies identified in the present thorough literature search were published within the last three years. Although the overall number of patients was reasonably large, the present results should be viewed in the light of the limitations of individual studies and the fact that estimates based on a small number of studies could be compromised by heterogeneity and/or bias, particularly in non-randomized settings.

### Main findings

Based on the agreement of univariate and independent associations indicated by the pooled estimates (and individual studies), the present data suggest that high on-admission SUA predicts higher short-term and medium/long-term mortality and incidence of MACE in AMI patients. “High” SUA appears to be in the range of hyperuricemia or high-normal values.

### Limitations of the individual studies

Most of the studies (6/9) were retrospective, and hence inherently susceptible to bias. Still, in the case of AMI, this limitation might not be particularly relevant as the condition is handled after pre-defined protocols with patient monitoring and accurate (real-time) data recording as a part of the good clinical practice standard, particularly considering short(er) time-periods (eg, during in-hospital stay). It is reasonable to expect susceptibility to bias/inaccuracy to increase with longer observational periods. All studies that reported on outcomes over periods longer than 30-day (8,9,11,13,14,16) were retrospective. However, two were based on databases generated prospectively after a pre-defined strategy (8,14), whereas methods implemented in the others (9,11,13,16) convincingly suggested a reasonably accurate identification of events and their timing. Hence, it seems plausible to consider individual study data as acceptably reliable.

No study matched the “high” and “low” SUA patients in respect to the major AMI outcome risk factors (eg, age, Killip class, or renal function) suggested by the established risk stratification systems (eg 22,23, ). Hence, although most of the multivariate models were quite complex, important effects or effect modifiers might have been occasionally omitted. While it seems unlikely that this fact could have resulted in misinterpretation of “no effect” or a “favorable effect” of high SUA as an “adverse effect” (particularly considering the qualitative agreement between univariate and multivariate estimates), it might have troubled the attempts to accurately quantify the true effect of high SUA.

### Strengths and limitations of the pooled analysis

The present literature search (no language or publication year restrictions) most likely omitted no relevant publication. The decision to exclude four of the identified prognostic studies was justified as they clearly referred to patients embraced in other included reports (17), were so poor in respect to reporting/methodology (18) that it was unclear whether they indeed dealt with the topic of interest, or included only sporadic AMI patients (20,21). The variability of the study particulars (AMI type/treatment, definition of MACE, consideration of SUA, follow-up duration, timing of the outcome assessment) further restricted the number of studies eligible for pooled analysis per outcome/time-period, particularly in the case of independent associations. We therefore addressed the issue of bias and, within the limitations of the available methodology, demonstrated that the observed SUA effects were not artifacts. Considering the methodological differences between studies (including differences in multivariate models), we a priori considered that no common effect size should be assumed and applied the random-effects meta-analysis. Still, for the short-term and simply defined outcomes, heterogeneity was non-existing or low and it increased when the composite outcome with variable definitions (MACE) or medium/long-term periods were considered. It should be noted, however, that heterogeneity was not due to essentially different study results (all individual unadjusted or adjusted estimates were in the same direction), but rather to the differences in size of the high(er) SUA effect. The undertaken exploration of heterogeneity should be viewed with caution, given the limited number of studies, but it indicates the disease/treatment characteristics (STEMI/PCI vs mixed STEMI/NSTEMI patients with variable reperfusion strategies) as its main source. Overall, the concordant primary random-effect and across-subset mixed-effect estimates provide evidence of an independent association of high(er) on-admission SUA and worse AMI outcomes that may vary in strength across the disease/treatment characteristics and the observed post-AMI period.

Some of the individual studies indicated that the effect of high SUA on medium/long-term outcomes was conditional on Killip class (more pronounced in patients with Killip class III/IV) (8), age (more pronounced in patients <63 years of age) (9), or on the levels of N-terminal pro-B-type natriuretic peptide (14) (more pronounced at higher concentrations). Since not based on the individual patient data, the present analysis could not address these issues.

### Practical relevance of the present observations

Considering that the individual studies were conducted in different settings reflecting daily situations and in different parts of the world that could differ in genetic/cultural factors affecting SUA (eg, nutritional habits, alcohol consumption, cigarette smoking), the present analysis supports a view about a robust association between the high(er) on-admission SUA and poor AMI outcomes. This observation has two potential practically relevant implications. First, it suggests that on-admission SUA should be considered in the risk stratification in AMI patients. In this respect, it should be noted that the independent effects of SUA (high-normal or hyperuricemic values) on short-term adverse outcomes are comparable in size to the effects of age >65 years, Killip class>II/III, or renal insufficiency, which are among the most important individual elements in the established risk stratification systems in AMI (22,23). Next, regardless of the known controversy about the mechanistic relationship between SUA and cardiovascular outcomes (a “direct pathogen” or a “mere marker” of the increased XO activity) (2-6), high on-admission SUA might depict situations that could benefit from XO inhibition. The results of a small placebo-controlled trial (19) showing beneficial short-term effect of allopurinol in STEMI/PCI patients support such a view.

### Conclusions

Over the past few years, several studies have evaluated the predictive value of on-admission SUA for the outcomes in AMI patients. Although still modest in number, the available data suggest that on-admission SUA in the high-normal or hyperuricemic range independently predicts worse short-term and medium/long-term outcomes. Additionally, preferably prospective studies are needed to more precisely quantify the relationship between SUA and the outcomes in different settings (eg, AMI type, reperfusion strategies) and to characterize the seemingly complex relationships between high SUA and other predictors.

## References

[1] Wu XW, Muzny D, Lee C, Caskey C (1992). Two independent mutational events in the loss of urate oxidase during hominoid evolution.. J Mol Evol.

[2] Pacher P, Nivorozhkin A, Szabo C (2006). Therapeutic effects of xanthine oxidase inhibitors: renaissance half a century after the discovery of allopurionol.. Pharmacol Rev.

[3] Feig DI, Kang D-H, Johnson RJ (2008). Uric acid and cardiovascular risk.. N Engl J Med.

[4] Lippi G, Montagnana M, Franchini M, Favaloro EJ, Targher G (2008). The paradoxal relationship between serum uric acid and cardiovascular disease.. Clin Chim Acta.

[5] Johnson RJ, Kang D-H, Feig DI, Kivlighn S, Kanellis J, Watanabe S (2003). Is there a pathogenetic role for uric acid in hypertension and cardiovascular and renal disesas?. Hypertension.

[6] Duan X, Ling F (2008). Is uric acid itself a player or a bystander in the pathophysiology of chronic heart failure?. Med Hypotheses.

[7] Kim SY, Guevara JP, Kim KM, Choi HK, Heitjan DF, Albert DA (2010). Hyperuricemia and coronary heart disease: a systematic review and meta-analysis.. Arthritis Care Res (Hoboken).

[8] Kojima S, Sakamoto T, Ishihara M, Kimura K, Miyazaki S, Yamagishi M (2005). Japanese acute coronary syndrome study (JACSS) investigators. Prognostic usefulness of serum uric acid after acute myocardial infarction.. Am J Cardiol.

[9] Car S, Trkulja V (2009). Higher serum uric acid on admission is associated with higher short-term mortality and poorer long-term survival after myocardial infarction: retrospective prognostic study.. Croat Med J.

[10] Lazzeri C, Valente S, Chiostri M, Sori A, Bernardo P, Gensini GF (2010). Uric acid in the acute phase of ST elevation myocardioal infarction submitted to primary PCI: its prognostic role and relation with inflammatory markers: a single center experience.. Int J Cardiol..

[11] Kowalczyk J, Francuz P, Swoboda R, Lenarczyk R, Sredniawa B, Golda A (2010). Prognostic significance of hyperuricemia in patients with different types of renal dysfunction after acute myocardial infarction treated with percutaneous coronary intervention.. Nephron Clin Pract.

[12] Lazzeri C, Valente S, Chiostri M, Picariello C, Gensini GF (2012). Uric acid in the early risk stratification of ST-elevation myocardial infarction.. Intern Emerg Med.

[13] Basar N, Sen N, Ozcan F, Erden G, Kanat S, Sokmen E (2011). Elevated serum uric acid predicts angiographic impaired reperfusion and 1-year mortality in ST-segment elevation myocardial infarction patients undergoing percutaneous coronary intervention.. J Investig Med.

[14] Bae MH, Lee JH, Lee SJ, Park SH, Yang DH, Park HS (2011). Serum uric acid as an independent and incremental prognostic marker in addition to N-terminal pro-B-type natriuretic peptide in patients with acute myocardial infarction.. Circ J.

[15] Akpek M, Kaya MG, Uyarel H, Yarlioglues M, Kalay N, Gunebakmaz O (2011). The association of serum uric acid levels on coronary flow in patients with STEMI undergoing primary PCI.. Atherosclerosis.

[16] Kaya MG, Uyarel H, Akpek M, Kalay N, Ergelen M, Ayhan E (2012). Prognostic value of uric acid in patients with ST-elevated myocardial infarction undergoing primary coronary intervention.. Am J Cardiol.

[17] Valente S, Lazzeri C, Vecchio S, Giglioli C, Mergheri M, Bernardo P (2007). Predictors of in-hospital mortality after percutaneous coronary intervention for cardiogenic shock.. Int J Cardiol.

[18] Homayounfar S, Ansari M, Kashani KM (2007). Evaluation of independent prognostic importance of hyperuricemia in hospital death after acute myocardial infarction.. Saudi Med J.

[19] Rentoukas E, Tsarouhas K, Tsitsimpikou C, Lazaros G, Deftereos S, Vavetsi S (2010). The prognostic impact of allopurinol in patients with acute myocardial infarction undergoing primary percutaneous coronary intervention.. Int J Cardiol.

[20] Spoon DB, Lerman A, Rule AD, Prasad A, Lennon RJ, Holmes DR (2010). The association of serum uric acid levels with outcomes following percutaneous coronary intervention.. J Interv Cardiol.

[21] Park SH, Shin WY, Lee EY, Gil HW, Lee SW, Lee SJ (2011). The impact of hyperuricemia on in-hospital mortality and incidence of acute kidney injury in patients undergoing percutaneous coronary intervention.. Circ J.

[22] Granger CB, Goldberg RJ, Dabbous O, Pieper KS, Eagle K, Cannon CP (2003). Predictors of hospital mortality in the Global registry of acute coronary events.. Arch Intern Med.

[23] Halkin A, Singh M, Nikolsky E, Grines CL, Tcheng JE, Garcia E (2005). Prediction of mortality after primary percutaneous coronary intervention for acute myocardial infarction. The Cadillac risk score.. J Am Coll Cardiol.

